# High-Precision Detection of Magnetic Nanoparticles in Microfluidic Biosensing Systems

**DOI:** 10.3390/bios16050291

**Published:** 2026-05-16

**Authors:** Dakota Brown, Wendell Manuel, Dan Luu, Tri-Duc Luong, Marienette Morales Vega, Manh-Huong Phan

**Affiliations:** 1 Department of Physics, University of South Florida, Tampa, FL 33620, USA; thorne3@usf.edu (D.B.); danluu@usf.edu (D.L.); ducluong1@usf.edu (T.-D.L.); 2 Materials Science and Engineering Program, College of Science, University of the Philippines Diliman, Diliman, Quezon City 1101, Philippines; wamanuel@up.edu.ph; 3 Center for Materials Innovation and Technology, VinUniversity, Hanoi 100000, Vietnam

**Keywords:** fluidic sensing, magnetic particles, copper coil detection

## Abstract

The low signal-to-noise ratio (SNR) of existing magnetic sensors limits the detection of magnetic nanoparticles (MNPs) in microfluidic biosensing. We present a novel microfluidic coil-based impedance detection system for quantifying magnetic particles, including Fe filings and citrate-coated Fe_3_O_4_ MNPs, with potential applications in magnetically guided biosensing. Unlike conventional approaches that directly measure the magnetic properties of dispersed particles, our method employs an external collector magnet to concentrate particles within a copper coil detector. The accumulated particles alter the coil’s electromagnetic response through changes in the sample’s dielectric properties, producing an amplified impedance signal proportional to sample volume. We evaluated detection performance for 1–10 mg of ferromagnetic Fe filings and citrate-coated Fe_3_O_4_ MNPs across a broad frequency range. Results show a strong linear correlation between particle mass and impedance change, with SNR values from 25 dB to over 45 dB, demonstrating high sensitivity and precision. Coil sensitivity was further optimized by varying the number of turns (5, 10, and 15), enabling frequency-specific customization. This approach provides a scalable, low-cost platform adaptable to polymer-coated MNPs targeting biological analytes.

## 1. Introduction

Detection of magnetic nanoparticles (MNPs) is crucial in biosensing because it enables highly sensitive, selective, and low-noise measurements in complex biological environments, supporting the development of advanced diagnostic tools and personalized medicine platforms [1,2,3,4,5,6]. MNPs can also be easily manipulated using external magnetic fields, making them ideal for microfluidic biosensors and lab-on-a-chip systems that aim for compact, automated, and fast diagnostics. Unlike optical or fluorescent methods, magnetic detection generally has minimal background noise from biological samples (like blood or serum), since biological tissues are diamagnetic or weakly paramagnetic [7]. This allows improvement in the signal-to-noise ratio (SNR) and measurement accuracy. An important advantage of MNPs is that they can be precisely manipulated and controlled using external magnetic fields. Magnetic forces can transport, concentrate, separate, or immobilize nanoparticles without requiring direct physical contact, making MNPs highly suitable for integration into microfluidic biosensors and lab-on-a-chip systems. In these platforms, magnetic manipulation can automate sample preparation steps such as mixing, washing, target capture, and separation, leading to compact, portable, and rapid diagnostic devices. Such capabilities are particularly valuable for point-of-care applications where fast analysis, low sample volume, and minimal user intervention are required.

In the realm of nanoparticle biosensing, superparamagnetic iron oxide nanoparticles (SPIONs), particularly Fe_3_O_4_, have emerged as versatile platforms due to their high magnetic susceptibility, biocompatibility, and ease of surface functionalization [8,9,10,11,12]. By coating these MNPs with polymers such as citrates or polyethylene glycol, they can be tailored to bind specific biological targets, such as proteins or pathogens, enabling selective detection in complex biological environments [13]. Consequently, enumerating such biomarkers has emerged as a salient field in recent years, promising far-reaching applications, from early disease detection to magnetically guided targeting [14]. To truly leverage the diverse operability of these MNPs, an equally diverse detection method that offers high sensitivity, high throughput, cost-efficient, easily interpreted, point-of-care measurements is required. Impedance-based detection systems offer a compelling approach due to their cheap, simple, and non-destructive means of quantification [15,16,17]. These systems consist of a sensing element that exhibits a remarkable change in resistance or reaction when the target agent is present. Prior studies have explored coil-based sensors for probing the magnetic signatures of functionalized MNPs [15,17].

Among magnetic transducers, magnetoimpedance (MI) [18] and giant magnetoimpedance (GMI) biosensors based on amorphous, soft ferromagnetic wires or ribbons have attracted considerable attention because they can exhibit very large impedance changes under small magnetic fields, enabling high sensitivity to magnetic nanoparticle labels [15,17]. Reported GMI-based platforms have demonstrated clear improvements in sensor response, typically quantified as the relative impedance change versus applied field or particle concentration, and have been successfully adapted to detect functionalized MNPs in liquid environments [15,16]. However, despite these advances in sensitivity, it remains unclear whether the overall SNR of GMI biosensors is also improved in practical biosensing conditions. Existing GMI studies rarely provide a systematic SNR analysis in terms of both signal amplitude and noise statistics [16]. Motivated by these SNR constraints, we explore an alternative strategy based on a simple copper coil operated as a high-frequency impedance sensor and contrast it with a GMI coil of matching dimensions. As a result, our platform routinely attains SNR values in the 25–45+ dB range for both ferromagnetic Fe filings and Fe_3_O_4_ MNPs, indicating a robust and quantifiable enhancement in SNR rather than sensitivity alone.

Magnetic biosensing using particle labels has been investigated over several decades [19,20]. Early work established the feasibility of detecting magnetic labels in biological environments using inductive and magnetoresistive techniques, with pioneering demonstrations appearing in the late 1990s. Subsequent studies explored microfluidic integration into magnetic detection platforms, including inductive sensors capable of quantifying surface concentrations of superparamagnetic microbeads, achieving detection limits on the order of 10^10^ Bohr magnetons [21]. These approaches generally rely on direct measurement of the magnetic moment or field perturbation induced by dispersed or immobilized particles.

In this study, we developed a novel microfluidic-compatible, coil-based impedance detection platform that concentrates magnetic particles (both ferromagnetic Fe filings and citric-coated Fe_3_O_4_ MNPs possessing zero remanence, zero coercivity, and high magnetic saturation) inside a simple copper coil using an external collector magnet. By measuring radio-frequency impedance perturbations (reactance X, resistance R, and impedance Z) caused by changes in the dielectric environment within the coil, rather than relying on direct magnetic signatures, the system achieves a significantly high signal without the cost of increased noise. Systematic frequency sweeps from 300 kHz to 3 GHz reveal a strong linear correlation between total particle mass (1–10 mg) and differential impedance (ΔZ), with SNR ranging from 25 dB to over 45 dB, which are superior to those obtained with identical GMI coils. Optimization of coil geometry through variation in the number of turns (5, 10, and 15) further enabled tunable operating frequencies while maintaining excellent SNR. This approach offers a simple, low-cost, and reversible alternative to MNP quantification, providing a promising foundation for the future integration into microfluidic biosensors capable of detecting polymer-functionalized MNPs conjugated with biological targets.

## 2. Materials and Methods

**Figure 1:**
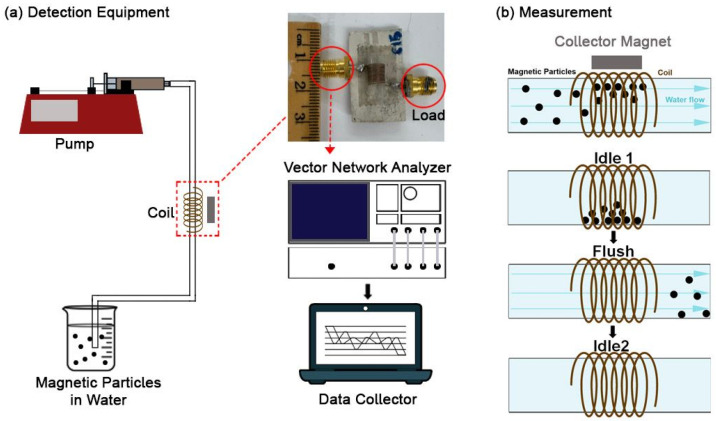
(**a**) Schematic illustration of the experimental setup for magnetic nanoparticle (MNP) detection using an Agilent HP 9753C Vector Network Analyzer (VNA) (Agilent Technologies, Santa Clara, CA, USA). The system includes a coil sensor (in dotted box, one end is connected to the VNA and another end on an SMA 50 Ω RF load), a pump operating at 20 mL/min, MNPs dispersed in water, and a collector magnet positioned at a distance of 1 mm to concentrate particles inside the coil sensor. (**b**) Schematic of how a measurement is performed. Particles are pumped through the tube and collected with the external collector magnet. Once removed, the parameters R, X, and Z are contrasted between the moments when particles are present and when they are not.

Figure 1 illustrates the experimental setup for MNP detection, which includes a vector network analyzer and a speed-controlled pump.

MNPs were concentrated within the coil using a bar magnet to attract and localize particles during fluid flow, followed by magnet removal to facilitate impedance measurements. The experimental protocol spanned 90 s, divided into three 30 s phases: an initial idle phase, a flush phase, and a final idle phase. During the initial idle phase, magnetic particles were retained within the coil, allowing stable measurement of baseline electromagnetic properties. In the flush phase, the pump was activated to clear the particles, leaving only water in the coil. The final idle phase provided a reference state with only water present. This method allows for selective frequency–sensitivity optimization of differing materials. Impedance parameters such as resistance (R), reactance (X), and total impedance (Z) were recorded using a vector network analyzer (VNA), which can sweep these parameters across frequencies up to 3 GHz. The differential parameters (∆R, ∆X, and ∆Z) were calculated as the difference between the average values of the first and second idle phases, reflecting the impedance perturbation induced by the presence and removal of magnetic particles. From the obtained measurements, the SNRs across the various frequencies were calculated and categorized from “not usable” to “excellent” (See Supplementary Information, Section S1: “Signal-to-Noise Ratio Definition” for more details). Frequencies exhibiting the highest SNR values (630 MHz for Fe filings and 600 MHz for Fe_3_O_4_ MNPs) were selected for operation. Citric-coated Fe_3_O_4_ MNPs were synthesized by the co-precipitation method as described in our previous study [22]. Unlike Fe filings, these Fe_3_O_4_ MNPs were found to exhibit superparamagnetic behavior at room temperature.

## 3. Results and Discussion

### 3.1. Sensor Design and Optimization

**Figure 2:**
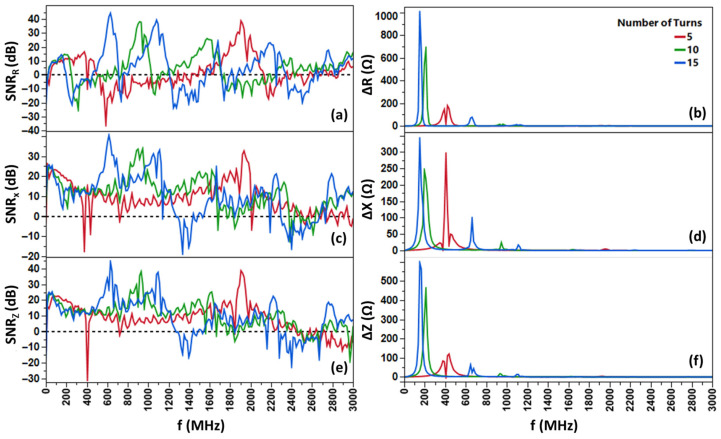
Frequency-dependent signal-to-noise ratio (SNR in dB) and absolute delta parameter values (∆R, ∆X, ∆Z in Ω) for copper coil sensors with varying numbers of turns (5 in red, 10 in green, 15 in blue), measured using 10 mg Fe filings. Left panels show SNR for (**a**) resistance, (**c**) reactance, and (**e**) impedance across frequencies from 0 to 3 GHz, demonstrating shifts in peak SNR to lower frequencies with increasing turn count. Right panels display corresponding delta parameters ((**b**) ∆R, (**d**) ∆X, (**f**) ∆Z), highlighting larger delta magnitudes at resonant frequencies that correlate with higher SNR peaks. Data was processed with MATLAB R2023a.

To enhance the sensitivity of our detection system, we investigated the effect of coil turn count on impedance measurements, a key factor in electromagnetic sensors like this one. We fabricated copper coils with 5, 10, and 15 turns that feature the same inter-turn spacing and tested them using a fixed 10 mg sample of Spacecare’s iron (Fe) filings to ensure fair comparisons. As shown in Figure 2, turn count correlates with higher sensitivity (45+ dB for a 15-turn coil) in conjunction with lower circuit resonant frequencies. Altering the number of turns provides a powerful avenue for flexible frequency accommodation towards varying system limitations. We see the ideal operational frequency shift from 600 MHz at 15 turns (45 dB) to 900 MHz at 10 turns (39 dB) to 1.9 GHz at 5 turns (38 dB).

The geometrical parameters of the copper coil prototypes are important because they define the effective probe size, sensing volume, and the proximity between the concentrated particles and the sensing region, all of which influence the impedance response and signal-to-noise ratio. In the current design, the copper coils were wound around the tube with an outer diameter of 4 mm and an inner diameter of 2 mm. The wire was an uninsulated bare copper wire with a diameter of 0.25 mm. The 5-turn coil was 2 mm, while the 10-turn coil was 4 mm, and the 15-turn coil was 6 mm, giving an axial pitch of about 0.4 mm per turn and an inter-turn spacing of approximately 0.15 mm. Because the turn spacing and overall coil diameter were kept constant, increasing the number of turns primarily increased the coil height and sensing length. These prototype dimensions are directly relevant to biosensing performance because they govern field confinement, sensing volume, and particle accumulation within the active detection region.

Turn-based sensitivity enhancement is backed by first principles: more turns increase inductance (scaling with the square of turns) and inter-turn parasitic capacitance, altering resonant frequencies. At higher frequencies (>200 kHz), the capacitance dependence of the system begins to dominate the change in impedance, much like in magnetoimpedance-based nanoparticle detectors [15,16,17].

**Figure 3:**
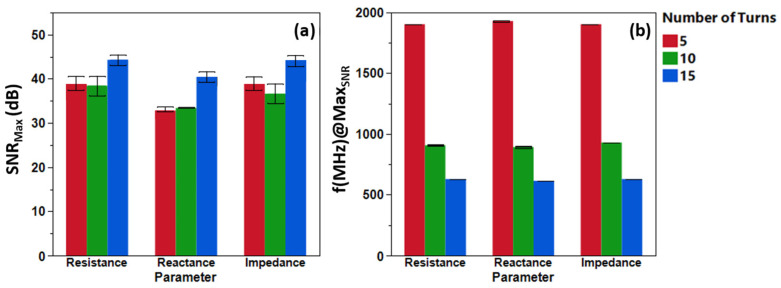
(**a**) SNR_max_ and (**b**) total parameter change for the 5-, 10-, and 15-turn coils collected at the center. The sample could not fit within the coil dimensions of the 5- and 10-turn coils, and therefore, not all of it was detected. Meanwhile, for the 15-turn coil, the maximum change was detected. Furthermore, an increasing number of turns also led to higher capacitive parasitic behavior. This was observed from the larger negative shift between the initial and final idle values in the X profile.

By increasing the number of turns, the operational frequency, which is defined as the frequency at which the maximum SNR occurs, also changes. As the number of turns increases, the operational frequency decreases, but the maximum SNR remains in a “very good” to “excellent” range (Figure 3). The operational frequencies of each coil are beyond their self-resonant frequencies, with an observable increase in the number of SNR peaks within the working frequency range as the number of turns increases. This is indicative that the system behaves like a transmission line rather than a simple coil [19] (See Supplementary Information, Section S3: “Transmission Line Behavior”).

To evaluate the sensing performance of different coil materials, we compared the sensitivity of a conventional 10-turn copper coil with that of a 10-turn coil fabricated from amorphous Co_69.25_Fe_4.25_Si_13_B_12.5_Nb_1_ giant magnetoimpedance (GMI) microwire, which had previously been employed for differentiating signals between nanoparticles (see Supplementary Information, Section S4: “Copper Coil vs. GMI Coil”). The comparison was performed across a nanoparticle sample mass range of 0.5–5 mg. The experimental results demonstrate that the copper-based coil consistently achieves a higher SNR_max_ than the GMI microwire coil for all tested sample masses. This improved performance indicates that the copper coil provides stronger and cleaner signal detection under the present measurement conditions. Although GMI materials are known for their high magnetic sensitivity and are often advantageous in magnetic field sensing applications, the simpler copper coil configuration exhibited superior overall detection capability for nanoparticle enumeration in this study. The enhanced SNR performance of the copper coil may be attributed to several factors, including reduced intrinsic magnetic noise, improved electrical stability, and more efficient inductive coupling with the magnetic nanoparticle signals. In contrast, the magnetic characteristics of the GMI microwire, while beneficial for certain differential sensing applications, may introduce additional complexities such as nonlinear magnetic responses or increased background fluctuations that can limit overall signal clarity. These findings highlight the strong raw enumeration capability of a simple copper coil system for magnetic nanoparticle detection. The results suggest that, despite the advanced magnetic properties of GMI-based materials, conventional copper coils remain highly effective and practical sensing elements, particularly in applications where high signal-to-noise performance, simplicity, and reproducibility are critical.

### 3.2. Particle Detection

**Figure 4:**
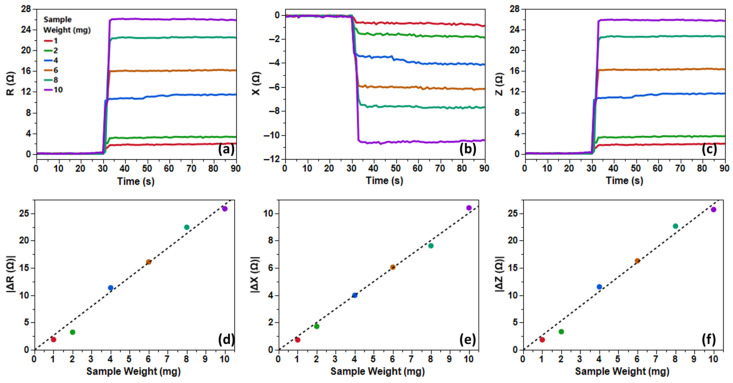
Time series plots of (**a**) resistance, (**b**) reactance, and (**c**) impedance for differing Fe filing sample weights (1, 2, 4, 6, 8, and 10 mg) at 630 MHz, as well as a linear fitting of the delta values (**d**–**f**).

The results for Fe filings (Figure 4) revealed a robust linear correlation between sample mass and ∆Z (R^2^ > 0.98), with SNR escalating from “good” (20–30 dB) at 1 mg—underscoring the system’s capability for milligram-scale detection—to “excellent” (40+ dB) at 10 mg, facilitating reliable quantification. Flushing restored baseline impedance, confirming reversible particle dynamics.

Ferromagnetic materials have attracted significant attention in biosensing technologies because of their strong magnetic properties and the ease with which their magnetic signals can be detected. One of the most important characteristics of ferromagnetic materials is their large remanent magnetization, meaning they are able to retain a considerable amount of magnetization even after the external magnetic field is removed. This persistent magnetic state generates a strong and stable magnetic signature, which can be readily detected by magnetic sensors with high sensitivity and reliability [23,24]. Owing to this property, ferromagnetic materials are widely investigated for use in magnetic detection platforms, signal amplification systems, and sensing devices where strong magnetic responses are advantageous. Despite these benefits, ferromagnetic materials also present several limitations when applied in biological and liquid environments. Their high magnetic moment causes strong dipole–dipole interactions between neighboring particles. As a result, the particles tend to attract one another and form aggregates or clusters. This magnetic agglomeration reduces their colloidal stability and makes it difficult to maintain a uniform dispersion in aqueous solutions or biological media such as blood, serum, or intracellular fluids. Aggregation can significantly impair sensing performance by reducing active surface area, altering magnetic behavior, and limiting the mobility of the particles. In biomedical applications, particle clustering may also lead to poor biocompatibility, reduced circulation time, and unwanted accumulation in tissues, thereby restricting the practical use of conventional ferromagnetic materials.

In contrast, Fe_3_O_4_ nanoparticles exhibit magnetic behavior that is much more suitable for biological applications. These nanoparticles typically demonstrate superparamagnetic properties at nanoscale dimensions, characterized by the absence of a coercive field and negligible remanent magnetization once the external magnetic field is removed. Because they do not retain permanent magnetization, the magnetic attraction between particles is greatly reduced, minimizing the tendency for agglomeration. This property allows Fe_3_O_4_ nanoparticles to remain well dispersed and form stable colloidal suspensions even in complex biological fluids. As a result, Fe_3_O_4_ nanoparticles see wider practical use in biomedical and biosensing applications such as targeted drug delivery, MRI contrast agents, and magnetic biosensors, where controlled dispersion and biocompatibility are critical requirements [25].

**Figure 5:**
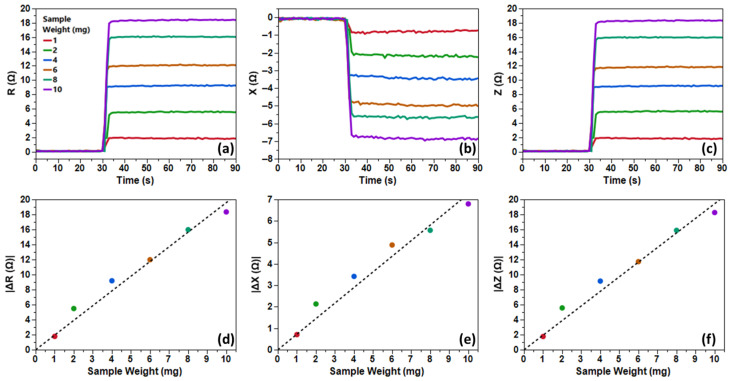
Time series plots of (**a**) resistance, (**b**) reactance, and (**c**) impedance for differing Fe_3_O_4_ MNP sample weights (1, 2, 4, 6, 8, and 10 mg) at 600 MHz, as well as a linear fitting of the delta values (**d**–**f**).

As one can see clearly in Figure 5, Fe_3_O_4_ nanoparticles exhibited linearity (R^2^ > 0.97) but with modestly attenuated ∆Z magnitudes. SNR progression mirrored the filings, advancing to “excellent” (40+ dB) at higher masses, indicative of the system’s viability to further test the Fe_3_O_4_ MNPs with linkers.

**Figure 6:**
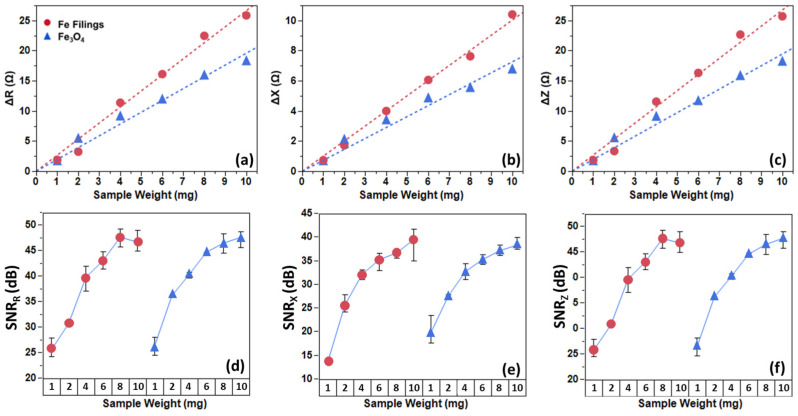
(**a**–**c**) Delta changes in both Fe filings and Fe_3_O_4_ MNP, as well as (**d**–**f**) SNR values for differing sample weights. Different slopes can be understood as a difference in dielectric properties.

Mechanistically, the observed impedance shifts stem from alterations in the dielectric material inside the coil, perturbing the electromagnetic field between turns in the coil, which exhibits high parasitic capacitance [26]. Despite exhibiting zero remanence and zero coercivity, we can still quantify the presence of Fe_3_O_4_ MNPs by probing their dielectric properties. Due to this dielectric-focused approach, a difference in slope on the sensors’ electromagnetic response at different weights between the Fe filings and citric-coated Fe_3_O_4_ MNP (Figure 6a–c) was observed. This opens up the possibility of the sensor being utilized for differentiation of not only magnetic particles but also attached biomarkers that possess different dielectric properties [27]. Despite the difference in the deltas, the MNPs show a comparable signal to the ferromagnetic Fe filings at equivalent masses, validating the platform’s versatility (Figure 6d–f).

This approach slightly modifies the established nature of impedimetric biosensors. In contrast to approaches that rely on the magnetic signature of functionalized MNPs for detection, we achieve higher specificity than traditional methods for point-of-care applications, yielding instantaneous results through non-destructive means. Limitations include frequency-dependent noise and minimum detectable concentrations (1 mg in our current setup), which could be mitigated by advanced coatings (e.g., silica for improved stability) or integration with microfluidic channels [28].

Future prospects aim to integrate this method into microfluidic channels. Shrinking the coil to the microfluidic regime (outer diameters typically on the scale of 0.3–2 mm and trace widths of 10–100 μm) will shift the frequency of higher-order transmission-line resonances upward due to the decrease in electrical length. In response to this, coils with larger turn counts and greater length may be manufactured to keep operational frequencies in the typical RF range (between 1 and 3 GHz). Miniaturization will improve the detection limit and SNR through closer proximity of the particles to the sensing element. Coil production in the microfluidic regime can be achieved with photolithography, electroplating, or wire-bonding techniques. Examples include wire-bonded solenoidal microcoils integrated with PDMS channels for on-chip NMR [29] and microcoils used for magnetic bead trapping and impedance-based detection in microfluidic chips [30]. By optimizing the geometry, the detection limit can be pushed towards sub-microgram levels while preserving high SNR values and maintaining a point-of-care status.

Fe_3_O_4_ MNPs are ideally suited for polymer coating, such as polyethylene glycol (PEG), chitosan, or polydopamine, to enhance colloidal stability, reduce cytotoxicity, and facilitate conjugation with biorecognition elements like antibodies, aptamers, or peptides [31]. For instance, PEG-coated Fe_3_O_4_ can be functionalized with target-specific ligands to bind biological entities (e.g., tumor biomarkers, pathogens, or antibiotics), forming MNP–polymer–target complexes that retain magnetic responsiveness for coil-based accumulation and detection [32]. In such scenarios, the impedance change would not only reflect MNPs but also the added dielectric properties of the bound target, enabling label-free, quantitative biosensing with limits of detection potentially reaching sub-microgram levels upon optimization if utilized inside microfluidic channels. Some reported biosensors, for example, rely on optical changes induced by biomolecular interactions at anisotropic interfaces [29]; the present work introduces a fundamentally different detection paradigm based on high-frequency impedance perturbations in a copper coil. Rather than probing molecular ordering, our approach exploits dielectric modulation produced by magnetically concentrated nanoparticles, enabling direct electrical quantification with optimized signal-to-noise ratios exceeding 45 dB. This decoupling of magnetic manipulation from electrical sensing provides a simpler, scalable, and instrumentation-independent alternative to optically driven sensing platforms.

## 4. Conclusions

We have demonstrated that a simple copper coil impedance-based system can function as a powerful tool for different magnetic materials. By probing the dielectric properties of the materials and using their magnetic nature to aggregate the particles inside the coil, we sidestep common struggles that occur when attempting to detect magnetic nanoparticles. We identified resonant operating points by calculating the signal-to-noise ratio for all operating frequencies. Operating conditions fall between SNR values of 25 to 48 dB, confirming high sensitivity at these mass ranges. Our system exhibits a linear response to sample mass over the tested range of 1–10 mg, as well as complete reversibility between flush states and a non-destructive means of nanoparticle quantification. We observe that in terms of nanoparticle quantification, a copper coil shows greater SNR values than a conventional GMI coil.

We understand our setup through the lens of a high-frequency transmission line whose parasitic capacitive elements are perturbed by the nanoparticles when they are inside the coil. The external magnet aims to maximize this effect by concentrating the nanoparticles as close to the coil as possible. This decoupling of magnetic manipulation from electrical detection enables a compact, low-cost geometry that is straightforward to optimize and inherently compatible with microfluidic architectures. Looking forward, integrating this coil-based impedance sensor into microchannels and combining Fe_3_O_4_ nanoparticles with biochemical linkers will allow further optimization of the demonstrated high SNR performance for selective biosensing of clinically relevant targets. Further work will focus on reducing the detectable mass, exploring microfabricated coil designs, and performing detailed SNR benchmarking under realistic flow and environmental conditions, positioning this approach as a promising alternative to more complex magnetic transducers for next-generation point-of-care diagnostics.

## Data Availability

The datasets generated and/or analyzed during this study are available from the corresponding author upon reasonable request.

## Supplementary Materials

The following supporting information can be downloaded at https://www.mdpi.com/article/10.3390/bios16050291/s1, Section S1: Signal-to-Noise Ratio Definition, Section S2: Synthesis of Citric-Coated Fe_3_O_4_ Nanoparticles, Section S3: Transmission Line Behavior and Section S4: Copper Coil vs. GMI Coil. Figure S1: FTIR of the synthesized Fe_3_O_4_ showing the effective coating of the citrate; Figure S2: The room-temperature M–H curve of the synthesized Fe_3_O_4_ nanoparticles exhibits zero coercivity and zero remanence. Together with the high magnetic saturation, these properties are consistent with the behavior of previously reported superparamagnetic Fe_3_O_4_ NPs at room temperature; Figure S3: Frequency-dependent signal-to-noise ratio (SNR in dB) and absolute delta parameter values (∆R, ∆X, ∆Z in Ω) for a copper coil sensor measured with varying sample weights of iron filings (1, 2, 4, 6, 8, and 10 mg); Figure S4: Frequency-dependent signal-to-noise ratio (SNR in dB) and absolute delta parameter values (∆R, ∆X, and ∆Z in Ω) for a copper coil sensor measured with varying sample weights of Fe_3_O_4_ MNPs (1, 2, 4, 6, 8, and 10 mg); Figure S5: Comparison of (a) resistance, (b) reactance, and (c) impedance SNRs of coils made from copper and GMI wire at different Fe_3_O_4_ sample weights. Each wire was made with 10 turns; Table S1: Classification of SNR values.

## Abbreviations

The following abbreviations are used in this manuscript:

MNP	Magnetic Nanoparticle
SPION	Super Paramagnetic Iron Oxide
GMI	Giant Magnetoimpedance
R	Resistance
X	Reactance
Z	Impedance
∆R	Differential Resistance
∆X	Differential Reactance
∆Z	Differential Impedance
VNA	Vector Network Analyzer
