# Supplementary material for: High-Precision Detection of Magnetic Nanoparticles in Microfluidic Biosensing Systems
Source: Biosensors (Basel). 2026 May 16;16(5):291. doi: 10.3390/bios16050291 (PMC13204941; doi:10.3390/bios16050291)
Supplement: Supplementary file 1 [file biosensors-16-00291-s001.zip › biosensors-4222645-supplementary.pdf]

Supplementary Information

High-Precision Detection of Magnetic Nanoparticles in Microfluidic Biosensing Systems

Dakota Brown [1,†], Wendell Manuel [2,†], Dan Luu [1], Tri-Duc Luong [1], Marienette Morales Vega [2,*] and Manh-Huong Phan [1,3,*]

[1] Department of Physics, University of South Florida, Tampa, FL 33620, USA; thorne3@usf.edu (D.B.); danluu@usf.edu (D.L.); ducluong1@usf.edu (T.-D.L.)
[2] Materials Science and Engineering Program, College of Science, University of the Philippines Diliman, Diliman, Quezon City 1101, Philippines; wamanuel@up.edu.ph
[3] Center for Materials Innovation and Technology, VinUniversity, Hanoi 100000, Vietnam
* Correspondence: mvega@msep.upd.edu.ph (M.M.V.); phanm@usf.edu (M.-H.P.)
† These authors contributed equally to this work.

S1. Signal-to-Noise Ratio Definition

Equation for Signal-to-Noise Ratio in dB:

$$SNR_{\Delta}(dB) = 20\log_{10}\frac{|\mu_{final}-\mu_{initial}|}{\sqrt{\sigma_{initial}^2+\sigma_{final}^2}} \tag{S1}$$

where SNR_{Δ} is the Signal-to-Noise Ratio in dB, $\mu_{initial}$ is the average of the parameter values on the first 20s, μ_{final} is the average of the parameter values on the last 20s, $\sigma_{initial}$ is the standard deviation of the parameter values on the first 20s,σ_{final} is the standard deviation of the parameter values on the last 20s. The change in R, X, and Z were also calculated by taking the difference between their respective μ_{final} and $\mu_{initial}$.

While there is no universally established guideline for classifying signal-to-noise ratio (SNR) values—as these vary depending on the application—a general rule of thumb presented in the table was applied in the context of this paper. The current experiment involves macroscopic amounts of nanoparticles for detection. This SNR range can be adjusted in future tests focusing on biosensing, where very small changes must be detected.

Table S1: Classification of SNR values

SNR (dB)	General Classification	Remarks
<0	Not Useable	Noise is dominating
0-5	Very Poor	Delta is slightly more but has a high tendency to get lost in the noise.
5-10	Poor/Barely Useable	Delta is present but noise is still high. Information can still be extracted but with difficulty and a high error rate.

10-20	Low but still useable	Marginal delta. OK for rough detection that does not need high precision.
20-30	Good	Delta is clearly dominant and noise is minimal. Reliable detection and acceptable accuracy.
30-40	Very Good	High Quality signal with very low noise.
>40	Excellent	Pristine signal and with high precision.

S2. Synthesis of citric coated Fe_3O_4 nanoparticles

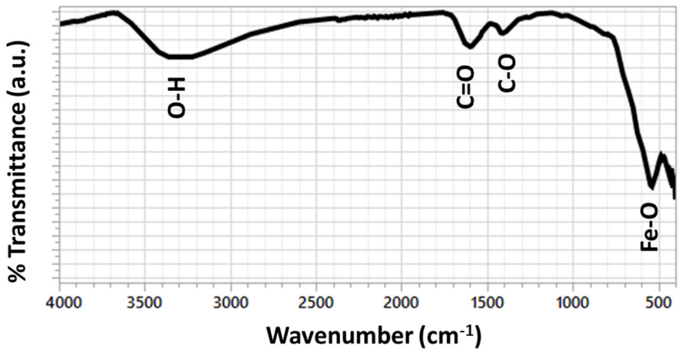

Figure S1. FTIR of the synthesized Fe_3O_4 showing the effective coating of the citrate.

Fe_3O_4 was synthesized as described on a previous study [22]. Fourier transform infrared spectroscopy (FTIR) confirmed the successful synthesis of citric acid–coated Fe_3O_4 (Figure S1). We associate the following peaks with the following bonds: 540 cm^{-1} Fe-O Stretch Sites from the Fe_3O_4, 1410 cm^{-1} from the C-O and 1602 cm^{-1} C=O stretch associated with the interaction between the coated citrate and Fe_3O_4, and 3302 cm^{-1} O-H stretch possibly coming from both Citric Acid and Fe_3O_4.

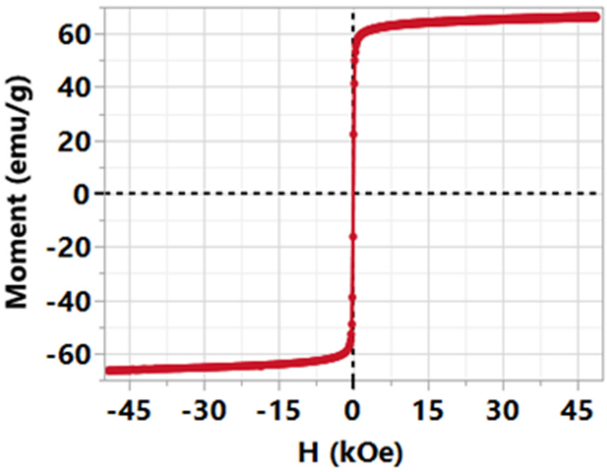

Figure S2. The room-temperature M–H curve of the synthesized Fe_3O_4 nanoparticles exhibits zero coercivity and zero remanence. Together with the high magnetic saturation, these properties are consistent with the behavior of previously reported superparamagnetic Fe_3O_4 NPs at room temperature.

S3. Transmission Line Behavior

Figure S and **Figure S** show the SNRs vs Frequency of Iron Filings and Fe_3O_4 at different sample weights, respectively. Both exhibit multiple frequencies with good to excellent SNR values. The presence of these peaks could indicate that, at high frequencies, the system behaves more like a transmission line rather than a simple coil, where the input signal propagates as a wave of finite velocity across the conductor [26] When the wavelength becomes a significant fraction of the effective length l_{eff} of the copper coil, resonances occur. The nth resonance frequency at the capacitive/transmission regime is given by the equation:

$$f_n = \frac{n v_p}{2 l_{eff}} = \frac{n}{2 l_{eff}\sqrt{L'C'}} \tag{S2}$$

where f_n is the nth-order higher resonance frequency, v_p is the phase velocity of the input signal, L' is the inductance per unit length, and C' is the capacitance per unit length. The effective length increases

with the number of turns, resulting in a lower first higher-order resonance frequency, which was observed. The equation shows that f_n is proportional to an integer multiple of n. This means that within any chosen frequency range, the number of possible f_n values increases in step with n.

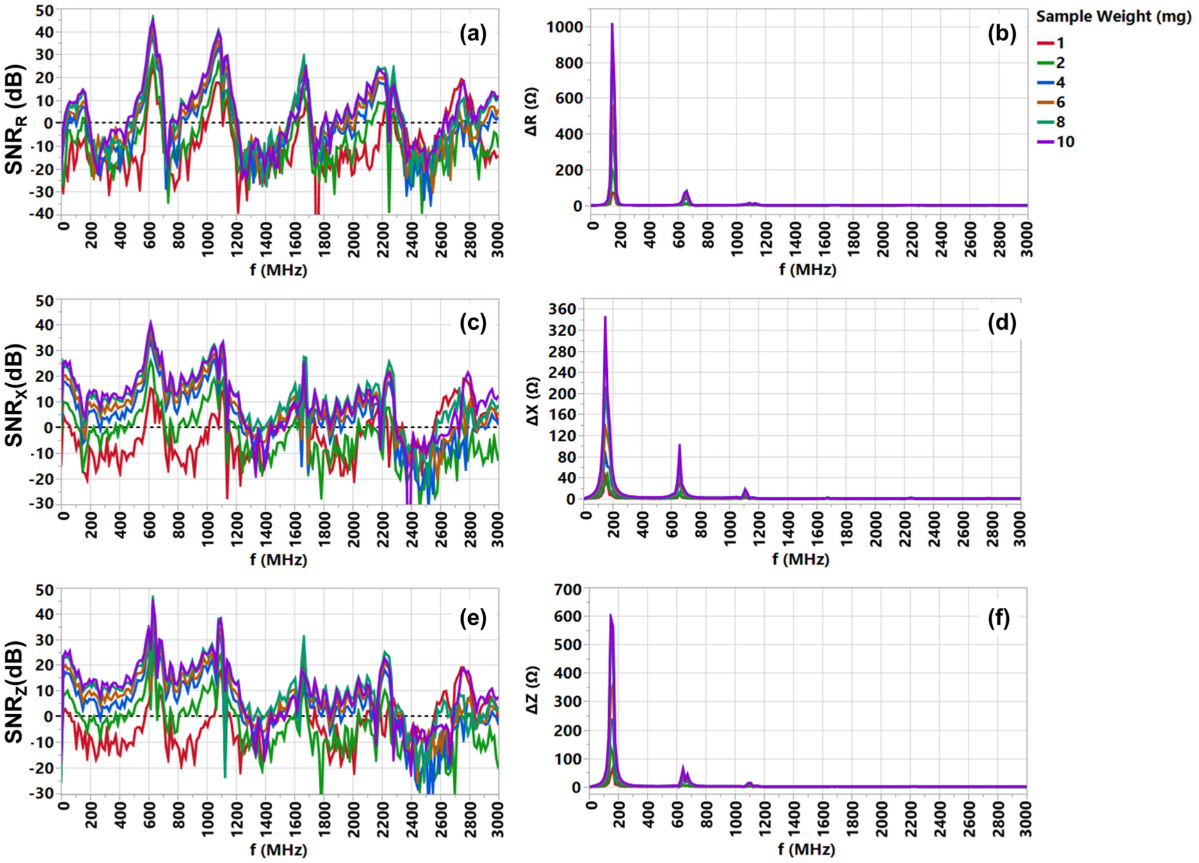

Figure S3. Frequency-dependent signal-to-noise ratio (SNR in dB) and absolute delta parameter values (ΔR, ΔX, ΔZ in Ω) for a copper coil sensor measured with varying sample weights of iron filings (1, 2, 4, 6, 8, 10 mg).

Left panels display SNR for resistance (top), reactance (middle), and impedance (bottom) across frequencies from 0 to 2000 MHz. Right panels show corresponding delta parameters (ΔR top, ΔX middle, ΔZ bottom) versus frequency, with larger magnitudes at resonant frequencies that scale linearly with sample weight. Optimal operational frequency is approximately 630 MHz for maximum SNR in Fe filings detection

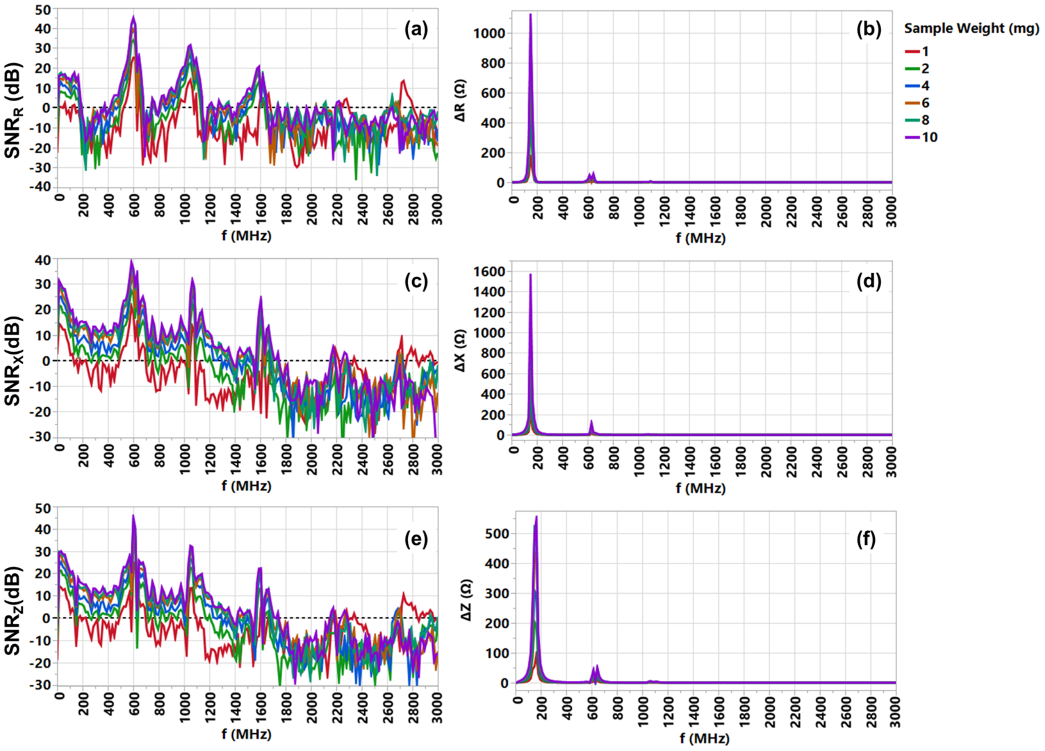

Figure S4. Frequency-dependent signal-to-noise ratio (SNR in dB) and absolute delta parameter values (ΔR, ΔX, ΔZ in Ω) for a copper coil sensor measured with varying sample weights of Fe_3O_4 MNP's (1, 2, 4, 6, 8, 10 mg).

Left panels display SNR for resistance (top), reactance (middle), and impedance (bottom) across frequencies from 0 to 2000 MHz. Right panels show corresponding delta parameters (ΔR top, ΔX middle, ΔZ bottom) versus frequency, similarly to the iron filings Fe_3O_4 Also shows linearly scaling changes in R, X, and Z with respect to total concentrated sample weight. Optimal operational frequency is approximately 600 MHz for maximum SNR in Fe_3O_4 detection.

S4. Copper Coil vs GMI Coil

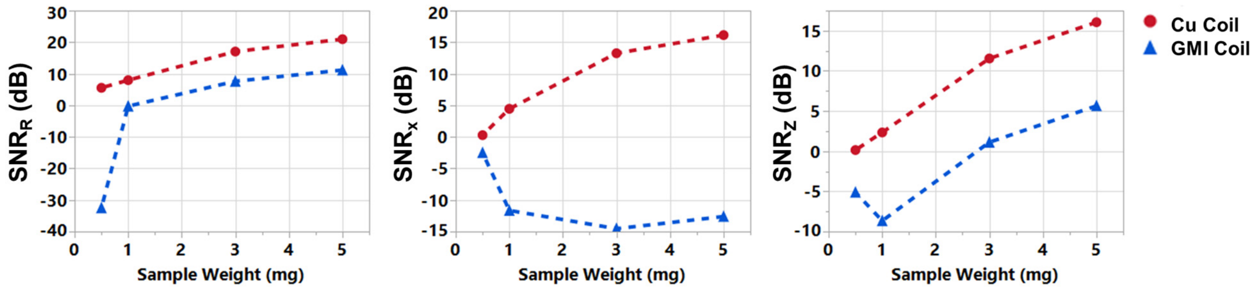

Figure S5. Comparison of (a) Resistance, (b) Reactance, and (c) Impedance SNRs of coils made from Copper and GMI Wire at different Fe_3O_4 sample weights. Each wire was made with 10 turns.

Results show that the coil made of Cu wire gave consistently better SNR values on all parameters across all sample weight.
